# Functional Connectivity Alterations in Epilepsy from Resting-State Functional MRI

**DOI:** 10.1371/journal.pone.0134944

**Published:** 2015-08-07

**Authors:** Kashif Rajpoot, Atif Riaz, Waqas Majeed, Nasir Rajpoot

**Affiliations:** 1 College of Computer Science & Information Technology, King Faisal University, Al Ahsa, Kingdom of Saudi Arabia; 2 School of Electrical Engineering and Computer Science, National University of Sciences & Technology, Islamabad, Pakistan; 3 School of Science and Engineering, Lahore University of Management Sciences, Lahore, Pakistan; 4 Department of Computer Science & Engineering, Qatar University, Doha, Qatar; 5 Department of Computer Science, University of Warwick, Coventry, United Kingdom; Institute of Psychology, Chinese Academy of Sciences, CHINA

## Abstract

The study of functional brain connectivity alterations induced by neurological disorders and their analysis from resting state functional Magnetic Resonance Imaging (rfMRI) is generally considered to be a challenging task. The main challenge lies in determining and interpreting the large-scale connectivity of brain regions when studying neurological disorders such as epilepsy. We tackle this challenging task by studying the cortical region connectivity using a novel approach for clustering the rfMRI time series signals and by identifying discriminant functional connections using a novel difference statistic measure. The proposed approach is then used in conjunction with the difference statistic to conduct automatic classification experiments for epileptic and healthy subjects using the rfMRI data. Our results show that the proposed difference statistic measure has the potential to extract promising discriminant neuroimaging markers. The extracted neuroimaging markers yield 93.08% classification accuracy on unseen data as compared to 80.20% accuracy on the same dataset by a recent state-of-the-art algorithm. The results demonstrate that for epilepsy the proposed approach confirms known functional connectivity alterations between cortical regions, reveals some new connectivity alterations, suggests potential neuroimaging markers, and predicts epilepsy with high accuracy from rfMRI scans.

## Introduction

Human brain can be envisaged as a complex functional network with brain cortical regions as nodes and functional connectivity between these regions as edges between the nodes. Each brain region is continuously sharing information with other regions thus resulting in a large-scale functional network [1]. Spontaneous low frequency fluctuations in resting-state functional Magnetic Resonance Imaging (rfMRI) data, collected while the subject is at rest, have recently been used to map functional connectivity non-invasively [2]. Recent studies on the analysis of rfMRI data have focused on large-scale brain connectivity [1, 3–5]. It has been shown [5–8] that brain disorders can cause disruption of the connectivity patterns amongst brain regions. Accurate identification of functional brain connectivity differences between patients and healthy subjects is crucial for understanding the functional effects of neurological disorders. The rfMRI has emerged as a powerful tool that has been employed in a number of studies to analyze functional connectivity alterations in the brain network for neurological disorders such as schizophrenia [9], epilepsy [8], Alzheimer’s disease [10, 11], and attention deficit hyperactivity disorder [5, 12].

In this work, we study the brain functional connectivity alterations caused by epilepsy using the resting-state fMRI data. Epilepsy is typically considered to be caused by abnormal neural activity in the cortex. This abnormal activity usually manifests itself through sudden excessive electrical discharge in neurons which can result in recurrent seizures. An epileptic seizure may bring changes in sensory perception, motor control, behavior, and/or autonomic function. Epilepsy affects about 50 million people in the world [13], 85% of whom live in developing countries, making it one of the most common neurological disorders. Recent research on rfMRI for epilepsy has focused on particular brain networks or regions of interest for a specific form of epilepsy. For instance, Zhang *et al*. [14] reported a decrease in the functional connectivity within dorsal attention network in mesial temporal lobe epilepsy (mTLE) patients. Their results indicated that top-down attention might be impaired in mTLE patients. Haneef *et al*. [15] demonstrated that the default mode network (DMN) might be altered in temporal lobe epilepsy (TLE). The DMN is involved in conscious and resting state cognition. Ji et al. [16] studied the functional alterations in mTLE subjects. As expected, they found connectivity changes in the mesial temporal lobe. Further, they identified specific epileptogenic zone (EZ) regions and showed that EZ demonstrated abnormal functional interaction with widespread brain regions throughout the brain. They concluded that the epileptic activity depends not solely on EZ, but it has relation with the activity emerging in macroscopic brain networks. In Ji et al. [17], the authors used rfMRI and diffusion tensor image scans to study inter-hemispheric functional and anatomical connectivity. They identified that the bilateral anterior cingulate cortex is critical to study the pathophysiology of patients with generalized tonic-clonic seizures in epilepsy. Chen *et al*. [18] studied alteration of mood regulation network in TLE patients with treatment-naïve depressive symptoms. Self-rating depression scale [19] was employed to evaluate depression symptoms of patients and hyperactivity in TLE patients was found in the prefrontal-limbic, striatal areas and anterior cingulate in patients with depressive symptoms (PDS). TLE patients of PDS group showed regional brain activity alterations and disruption of the mood regulation network at the onset of seizures.

The studies cited above provide valuable information about disease-related changes within particular brain networks. However, the potential of rfMRI for studying neurological disorders can be greatly enhanced when the information available in the data is utilized more fully by combining various discriminative neuroimaging markers in the form of connections in a machine learning framework. One way of achieving that would be to segment the brain into functional regions using a brain atlas and utilize inter-regional functional connectivity measures as features for classification. However, such an approach will suffer from the “curse of dimensionality” due to a large number of features, and a strategy to reduce the number of features and choosing the most relevant features (i.e. neuroimaging markers) needs to be incorporated in such a framework. Based upon these considerations, Zhang *et al*. [8] recently presented an algorithm for discrimination of epileptic and normal subjects from the rfMRI data. They employed *k*-means clustering for estimating functional brain connectivity. However, their work has following limitations: (a) the number of clusters must be specified *a priori*, making it less than ideal in domains like rfMRI where the precise number of clusters is unknown, and (b) the results of clustering are sensitive to initial arbitrary selection of centroids. To avoid the effect of dependence on initialization of centroids, Zhang *et al*. repeated the *k-*means algorithm with 500 random initializations and averaged the results to obtain a community matrix. The total number of clusters was somewhat arbitrarily chosen to be 30.

Our approach for connectivity analysis is based on a variant of affinity propagation (AP) clustering which does not require prior information about the number of clusters and does not involve arbitrary initial selection of centroids. AP clustering, proposed recently by Frey and Dueck [20], has been shown to perform well in multiple domains like clustering images of faces, gene expression and others [20]. Recent studies have effectively employed AP clustering in fMRI to study functional brain connectivity [21, 22]. Various studies have shown that AP clustering algorithm achieved low error rate as compared to *k*-means [21, 23].

In this paper, we explore the functional connectivity anomalies induced by epilepsy without limiting the scope to a particular brain network or to a specific type of epilepsy. For this purpose, we follow a three-stage approach. In the first stage, the functional connectivity between brain cortical regions is estimated by employing a variant of the AP clustering algorithm. In the second stage, we employ a novel difference statistic to enable the identification of discriminant connections between various cortical regions. In the third and final stage, we train a support vector machine (SVM) classifier to predict normal and epileptic subjects from their rfMRI scans. The main contributions of our work are as follows: (i) a method for intelligent initialization of the preference parameter of AP clustering to cluster functionally related brain regions into same groups, (ii) a novel difference statistic measure to enable identification of discriminative functional connections, and (iii) study functional connectivity alterations due to epilepsy in terms of brain lobes and the known resting state networks.

## Material and Methods

### Data

The rfMRI data used in our study is the same as in Zhang *et al*. [8] published earlier in PLOS ONE, which was acquired under approval by the local medical ethics committee (Jinling Hospital, Nanjing University School of Medicine, China) and a written informed consent was obtained from all patients. The data acquisition and pre-processing details are described here for the sake of completeness. The data contained 180 right-handed subjects including 80 healthy controls (age 24.89 ± 8.63) and 100 epileptic patients (age 23.85 ± 5.66). The epileptic subjects have different kinds of epilepsy (e.g., focal epilepsy, generalized epilepsy, temporal lobe epilepsy). There were 18 epileptic patients with generalized seizure and 70 with focal seizures. Antiepileptic drugs (AEDs) were used by 82 patients and 18 were not using any AEDs.

Before scanning, all the subjects were asked to relax, close their eyes without falling into sleep, hold still and think nothing in particular. The rfMRI data was collected on 3 Tesla Siemens Trio Tim scanner machine with an eight channel phase array head coil. Echo-planar imaging (EPI) sequence was used to acquire rfMRI data, with following parameters: TR/TE = 2000ms/30ms, FA = 90°, matrix = 64 × 64, FOV = 24 × 24cm^2^, slice thickness = 4mm, slice gap = 0.4mm and slices acquired = 30. Moreover, the routine anatomical MRI data was also acquired to study structural details. T1-weighted image scans were obtained, with following parameters: TR/TE = 350ms/2.46ms, FA = 90°, matrix = 320 × 256, FOV = 24 × 24cm^2^, slice thickness = 4mm, slice gap = 0.4mm, and acquired slices = 30. Coronal T2-FLAIR-weighted image scans were also obtained, with following parameters: TR/TE = 7000 ms/87 ms, FA = 150°, matrix = 256 × 256, FOV = 24 × 19.5cm^2^, slice thickness = 4mm, slice gap = 0mm and acquired slices = 28.

Data-preprocessing was performed using Data Processing Assistant for resting-State fMRI (DPARSF) software [24] which is based on Statistical Parametric Mapping (SPM) [25] and resting-state fMRI data analysis toolkit (REST) [26]. The pre-processing included slice timing adjustment and re-alignment for head motion correction. Spatial normalization was performed by using standard Montreal neurological institute (MNI) template provided by SPM2 (resampling voxel size: 3 × 3 × 3mm^3^). Subsequent to smoothing (with FWHM = 8mm), the BOLD signal was filtered in order to reduce low-frequency drift and high-frequency noise with band-pass = 0.01∼0.08Hz. Furthermore, the following variables were regressed out: (i) 6 parameters for head motion, (ii) global mean signal, (iii) white signal, and (iv) CSF signal. The automated anatomical labeling (AAL) template of Tzourio-Mazoyer *et al*. [27] was used to segment registered fMRI time series into 116 regions, 90 for cortex and 26 for cerebellum. The list of 90 cortical regions is given in S1 Table. Though the AAL parcellation is anatomical in nature, the regions thus identified are mapped to brain function. The AAL parcellation is commonly used in functional neuroimaging studies (e.g. [28, 29]). For each region, fMRI time series of all voxels lying in that region were averaged to obtain representative fMRI time series or BOLD signal of that region. Therefore, for each subject, there are 116 BOLD signals where *x*_*i*_(*t*) represents BOLD signal of the *i* ^th^ brain region. For this study, we focus at 90 brain regions belonging to cortex.

The head movement of the subject can influence the functional connectivity measurements. Thus, all the subjects were asked to control head movement such that there was only very small head movement (translation < 1mm and rotation < 1° for all subjects except two patients, while their rotations were less than 1.5°) which have been regressed out in preprocessing. Furthermore, two-sample *t*-test was performed for 6 parameters of head motion (3 parameters each for rotation and translation), for both the healthy and epileptic subjects, which showed no significant difference between the two groups.

### Determining Functional Connectivity

Functional connectivity between brain regions is typically calculated via temporal correlation between each pair of brain regions. To obtain a functional connectivity network having sparse connections, we propose a novel approach based on clustering of brain regions using the AP clustering algorithm. For the sake of completeness, below we give a brief description of the AP clustering.

AP clustering is a message passing algorithm where, unlike many clustering algorithms, there is no need for initial random selection of centroid and no need to specify the number of clusters. In this method, each data point is considered as a candidate for exemplar (centroid) and each data point is also considered initially as a candidate point belonging to all the clusters. In our context, the data point *i* can be considered as the BOLD time series *x*_*i*_ **= {** *x*_*i*_(*t*)**}** corresponding to the *i* ^th^ cortical region. During the iterative process of the algorithm, the messages are exchanged between the data points until robust exemplars (centroids) and clusters emerge. There are two kinds of messages which are exchanged between the data points: responsibility and availability. The responsibility message *r*(*i*, *j*) sent from the data point *i* to a candidate exemplar point *j* indicates how strongly *i* favors *j* to be chosen as its exemplar as given by (1). The availability message *a*(*i*, *j*) sent from the candidate exemplar point *j* to a point *i* indicates the extent to which *j* is available to become the cluster center (i.e. exemplar) for *i* as in (2). The availability message *a*(*i*, *j*) is initialized with 0 and updated as described below. These responsibility and availability messages are iteratively passed and updated between data points to determine cluster exemplars and clusters:

Responsibility message update:

$$r\left(i,j\right)=S\left(i,j\right)-\;\underset{j\prime ,j\prime \neq j}{\text{max}}\left\{a\left(i,j\prime \right)+S\left(i,j\prime \right)\right\}$$ (1)

Availability message update:

$$a\left(i,j\right)=\text{min}\left\{0,r\left(j,j\right)+\sum_{i\prime ,\;i\prime \neq \left\{i,j\right\}}\text{max}\left\{0,r\left(i\prime ,j\right)\right\}\right\}$$ (2)

where *S*(*i*, *j*) is the similarity measure between data points *i* and *j* computed as below:

$$S\left(i,j\right)=1-\frac{<x_{i}.x_{j}>}{\sqrt{<x_{i}.x_{i}>}\sqrt{<x_{j}.x_{j}>}}$$ (3)

and < *x*_*i*_.*x*_*j*_ > denotes the dot product of data points *x*_*i*_ and *x*_*j*_. An initial preference value *p* is assigned to each data point representing its probability of being a candidate exemplar (i.e. cluster centroid). The preference value *p* plays an important role in determining the cluster formations and the extraction of exemplars. Therefore, we propose a simple and intuitive method to initialize it. The preference value *p*(*i*) assigned to a data point *i* of being an exemplar is computed as:

$$p\left(i\right)=\;\mu \;\left(\underset{n}{\text{max}}\left(S(i,:)\right)\right)$$ (4)

where $\underset{n}{\text{max}}$ denotes choosing the *n* largest values from the vector *S*(*i*, :) which denotes the *i*th row vector of the similarity matrix *S*, and *μ*(.) denotes the mean. The value of *p*(*i*) computed using (4) denotes the average of similarity values of *n* most similar data points to *i*. Therefore, the value *p*(*i*) is an indicator of the likelihood of the data point *i* to become the exemplar in the subsequent clustering process.

**Fig 1:**
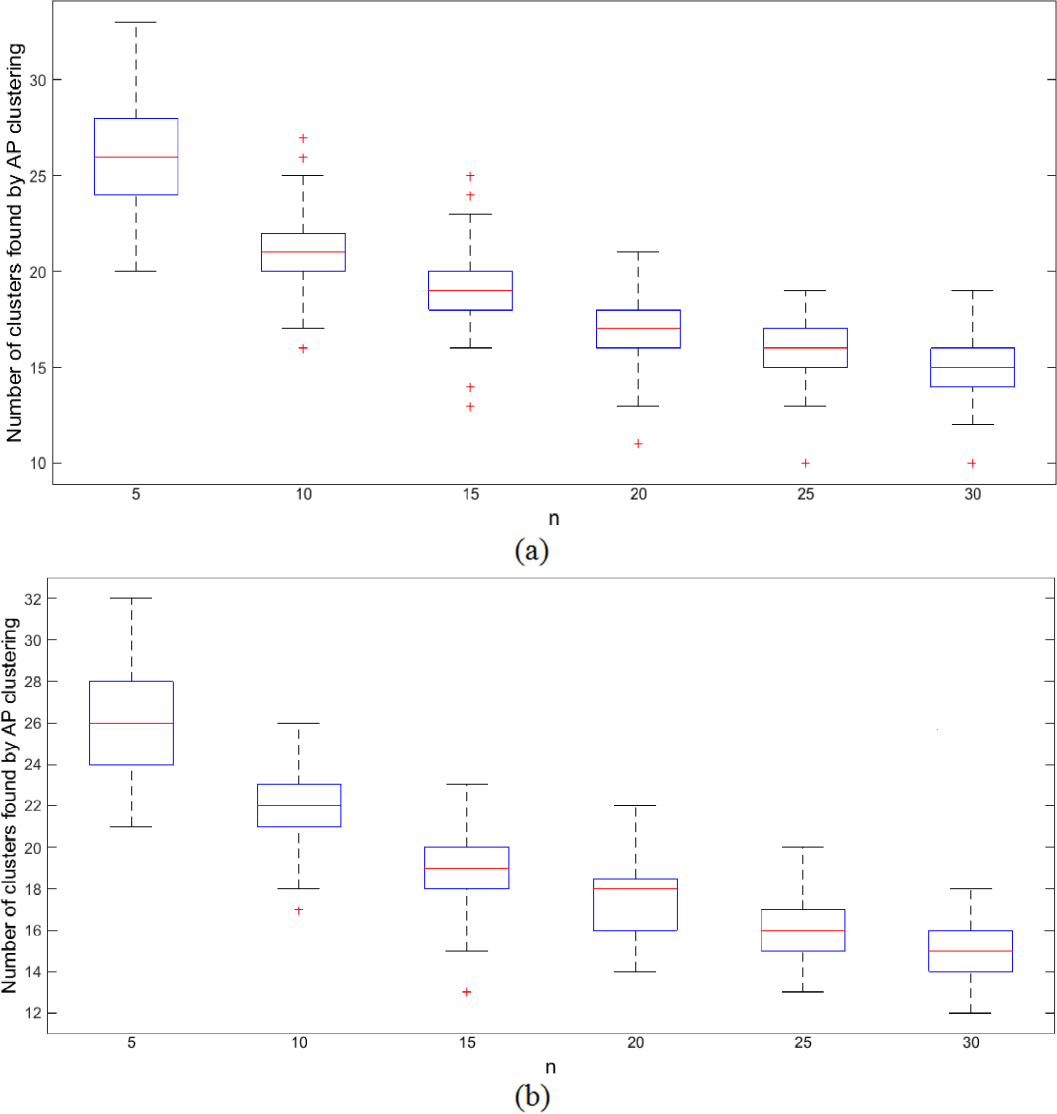
Relationship between the number of clusters found by AP clustering to the number of closest neighbors *n* in (4) used for computing the preference value *p*(*i*). (a) normal subject (80 in total), (b) epileptic patients (100 in total).

For a specific value of *n*, a connectivity matrix *C* is constructed such that *C*_*l*_(*i*, *j*) of brain regions *i* and *j* is calculated as:

$$C_{l}\left(i,j\right)=\;\{\begin{array}{ll} 1, & \;\text{if}\;i\;\text{and}\;j\;\text{are grouped in same cluster}\\ 0, & \;\text{otherwise} \end{array}$$ (5)

where *l* = 1,2, …, *L* is an index of the connectivity matrix generated by using a specific value of *n*, and *L* denotes the total number of connectivity matrices generated by using various different values of *n*. By varying *n* in (4), the preference value *p*(*i*) can be changed which can consequently affect the number of clusters produced. Therefore, experiments were conducted by varying *n* to test the consistency of functional connectivity among pairs of brain regions. In this way, we generate multiple connectivity matrices by varying *n* in (4) from 5 to 30 in steps of 5. It is worth noting that *n* denotes the number of most similar regions. The smaller the value of *n*, the higher the number of preference values *p*(*i*) that are relatively large, thus resulting in larger number of clusters (e.g., between 20 and 33 clusters for the 180 subjects in this study when *n* is 5). With a large value of *n* (e.g. 30), fewer preference values *p*(*i*) will be relatively large thus resulting in smaller number of clusters (between 10 and 21 clusters in our case). The plot of Fig 1 shows the number of clusters found in this way against the number of *n* closest regions used in computing the preference value *p*(*i*).

Through these multiple runs of clustering, *L* number of connectivity matrices *C* were obtained. From these connectivity matrices, we construct a community matrix *K* such that each *K*(*i*, *j*) is calculated as:

$$K\left(i,j\right)=\;\frac{1}{L}\sum_{l=1}^{L}C_{l}\left(i,j\right)$$ (6)

where *L* = 6 and *C*_*l*_ is the *l* ^th^ connectivity matrix. The value *K*(*i*, *j*) provides an estimate of the probability of *i*th and *j*th brain regions belonging to the same functional community network. The proposed approach can be considered as *multi-level clustering* where the number of clusters varies as the value of *n* changes in (4). A high value of *K*(*i*, *j*) shows that brain regions *i* and *j* were consistently clustered together in this multi-level clustering scheme, and therefore are more *likely* to be connected to each other.

**Fig 2:**
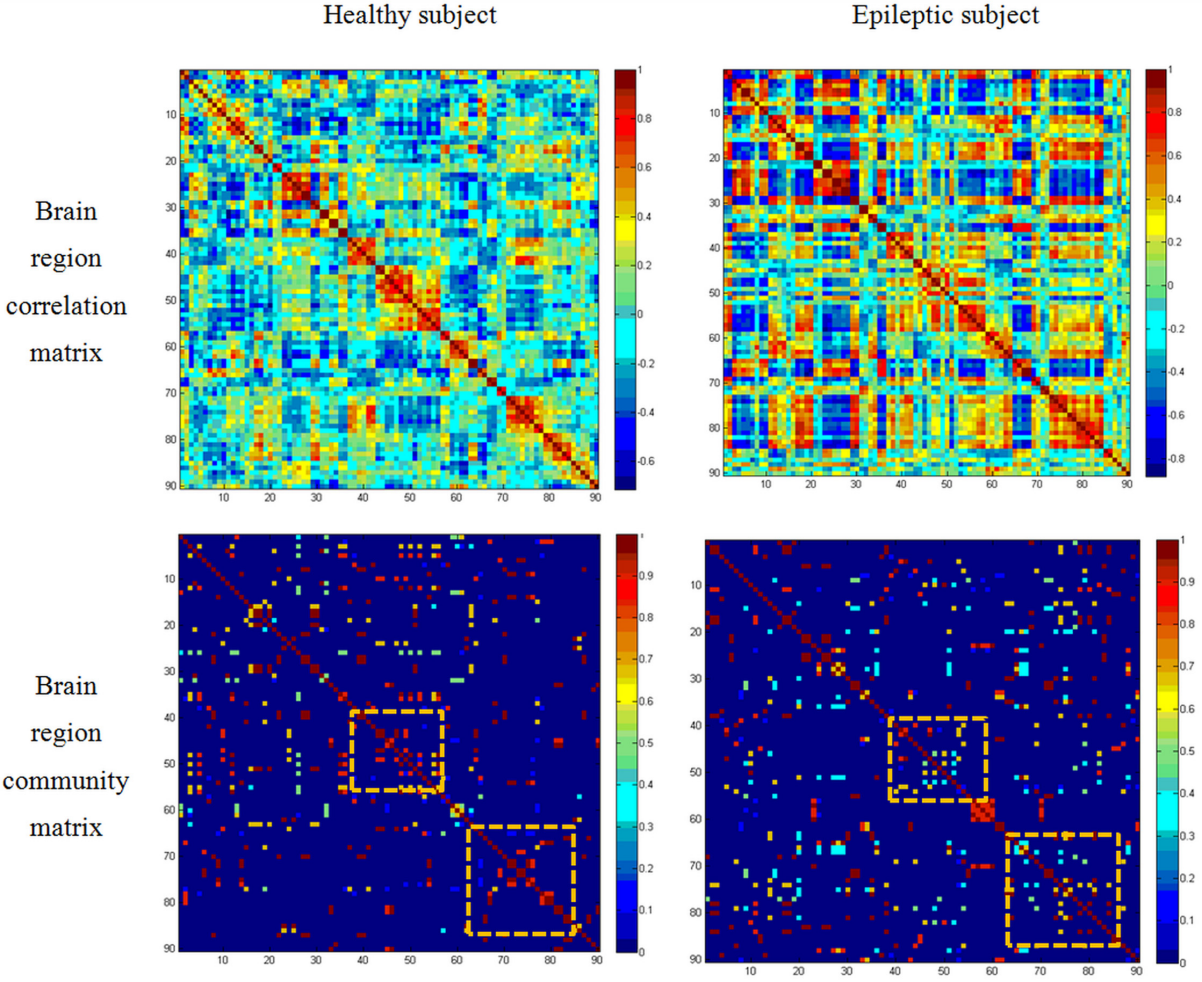
Brain region functional network: visualization of the correlation matrix [8] and community matrix obtained using (5). The difference between healthy and epileptic subjects is not prominent in the correlation matrix while it is prominent in community matrix (highlighted by boxes). This figure is suitable for visualization in color display.

The community matrix *K* represents functional brain network in a sparse manner which may not be possible with the typical correlation matrix. We can observe from Fig 2 that the connectivity differences between normal and epileptic subjects may be difficult to identify from dense data in the correlation matrix while the differences may be identified relatively easily from the sparse data in the community matrix.

The resultant community matrix *K* is a symmetric matrix containing functional connectivity information between every pair of brain regions *i* and *j*. Thus, community matrix *K* is an *N* × *N* matrix, where *N* = 90 in our case corresponding to 90 cortical regions. This community matrix *K* contains *N* × (*N* − 1)/2 distinct connections i.e. 4005 distinct connections in our case. The total number of distinct connections from the community matrix *K* seems be too high. These connections may include both discriminatory and non-discriminatory connections which may not necessarily help in our understanding of the underlying network connectivity. In this work, we aim to extract discriminative neuroimaging markers to address this so-called *curse of dimensionality* problem. We identify a smaller number of connections having high discriminatory power using the methods proposed in the next section.

### Identification of Discriminant Connections

We first construct a difference matrix *D* from the community matrices of epileptic and normal subjects such that the difference statistic *D*(*i*, *j*) reflects the discriminatory power of the undirected edge between the *i*th and *j*th brain regions. Each entry *D*(*i*, *j*) is calculated as:

$$D\left(i,j\right)=\;\left|\mu \left(K^{-}(i,j)\right)-\;\mu \left(K^{+}(i,j)\right)\right|$$ (7)

where *μ*(*K* ^−^(*i*, *j*)) denotes the mean of all normal community matrices *K* ^−^ at index (*i*, *j*) and *μ*(*K* ^+^(*i*, *j*)) denotes the mean of all patient community matrices *K* ^+^ at index (*i*, *j*). The difference matrix *D* reflects the discriminatory power of each connection–the larger the *D*(*i*, *j*) value, the higher the discriminatory ability. In this way, the difference matrix *D* represents the differences in functional connectivity between healthy and epileptic groups. The difference matrix *D* serves as a way to extract important neuroimaging markers for epileptic subjects based on the differences in their functional connectivity. In the next step, we exploit this potential by identifying discriminant connections.

We propose two methods for the identification of most discriminant connections which could be potentially used as neuroimaging markers. In both the methods, an iterative procedure selects the suitable discriminant connections. For each method, 100 iterations are run such that, for each iteration, the data is randomly divided by 50% − 50% split into training data and unseen test data. In each iteration, following steps are performed: (i) the top *h* connections with largest difference statistic value *D*(*i*, *j*) are selected from the training data, (ii) the SVM classifier is trained on the training data, and (iii) the performance of these selected connections is evaluated by validating the trained classifier on the test data. To test the dependency on the *h* selected connections, the value of *h* was varied from 350 to 600 in intervals of 50 (see the Results section for more details). The two proposed methods are described below:

1. *High-accuracy neuroimaging marker identification*: Let *c*_*i*_ denote the set of top *h* connections during the *i*th iteration. In this method, we select the set *dc*_*high*_ of discriminant connections which yielded the highest classification accuracy across all the 100 iterations as given by:
  $$dc_{high}=\;argmax\left[Acc{\left(c_{i}\right)}_{i\in \left\{1,2,\ldots ,100\right\}}\right]$$ (8)
  where *Acc*(*c*_*i*_) returns classification accuracy obtained with connections *c*_*i*_.
2. *Consistent neuroimaging marker identification*: Let *c*_*i*_ denote the set of top *h* connections during the *i*th iteration. Let $c={⋃}_{i=1}^{100}\;c_{i}$ denote the set of all distinct connections across all 100 iterations. Let *ω* denote the set consisting of values for frequency of occurrence across all the 100 iterations for each connection in *c*. Let $\tilde{c}$ denote the set of distinct connections in *c* but this time ordered according to their corresponding frequency values in *ω* sorted in a descending order. Then, the subset *dc*_*consistent*_ of *h* discriminant connections is selected by picking the first *h* connections in $\tilde{c}$ as follows:
  $$dc_{consistent}=\;\tilde{c}\left[1,2,\ldots \;,h\right]$$ (9)

The neuroimaging markers identified using one of the either two methods described above are provided to the next step of classification.

### Classification

The third and final step of the proposed connectivity analysis is the classification on the basis of discriminant connections. For this purpose, the support vector machine (SVM) [30] classifier with a linear kernel is employed for classification of normal and patient groups. The data was divided into two subsets: 50% for training and the remaining 50% for testing. We used the training data to train the SVM classifier. The unseen testing data was then presented to the SVM classifier. The performance of the classifier was assessed using the well-known measures of classification accuracy, specificity, and sensitivity.

## Results

### Experiments with High-accuracy Neuroimaging Marker Identification

We experimented by varying the number of *h* discriminant connections from 350 to 600, in order to observe the dependency of the classifier’s prediction ability upon selected connections. For each case, the experiment was repeated 100 times by randomly distributing the data into training and testing subsets to avoid any bias in experimental setup. The results presented in Table 1 show the average classification accuracy over 100 random cross-validation trials. The results indicate that the accuracy is not considerably affected by the variations in the number of selected connections. With 450 selected connections, average accuracy of 81.33% was achieved. These results demonstrate that the proposed method is able to identify sensitive neuroimaging markers which have the capability to accurately discriminate between normal and epileptic subjects.

**Table 1:** Classification accuracy with high-accuracy neuroimaging marker identification method.

Number of connections	Prediction accuracy	Specificity (mean)	Sensitivity (mean)
**300**	74.5%	68.9%	79.1%
**350**	80.2%	75.1%	84.3%
**400**	80.8%	74.5%	85.8%
**450**	81.3%	75.6%	85.9%
**500**	79.8%	73.9%	84.4%
**550**	79.0%	76.7%	80.8%
**600**	78.4%	71.7%	83.7%

For this experiment, 50% dataset is used as training and rest 50% as testing over 100 trials. It can be seen that best accuracy is attained with 450 connections.

### Comparison with Zhang et al. [8]

To evaluate the proposed method, we implemented the recently proposed work of Zhang *et al*. [8] which employs *k*-means clustering for community matrix construction, sparse regression for identification of discriminant connections, and SVM classification. Our results show that our proposed method outperforms the algorithm proposed in Zhang *et al*. [8] for the purpose of neuroimaging marker identification. With the proposed method, we achieved classification accuracy of 80.79% using 400 connections, while Zhang *et al*. [8] reported 77.60% accuracy using same number of connections. Note that both the studies are using the same rfMRI data, with identical split for training and testing subsets.

**Fig 3:**
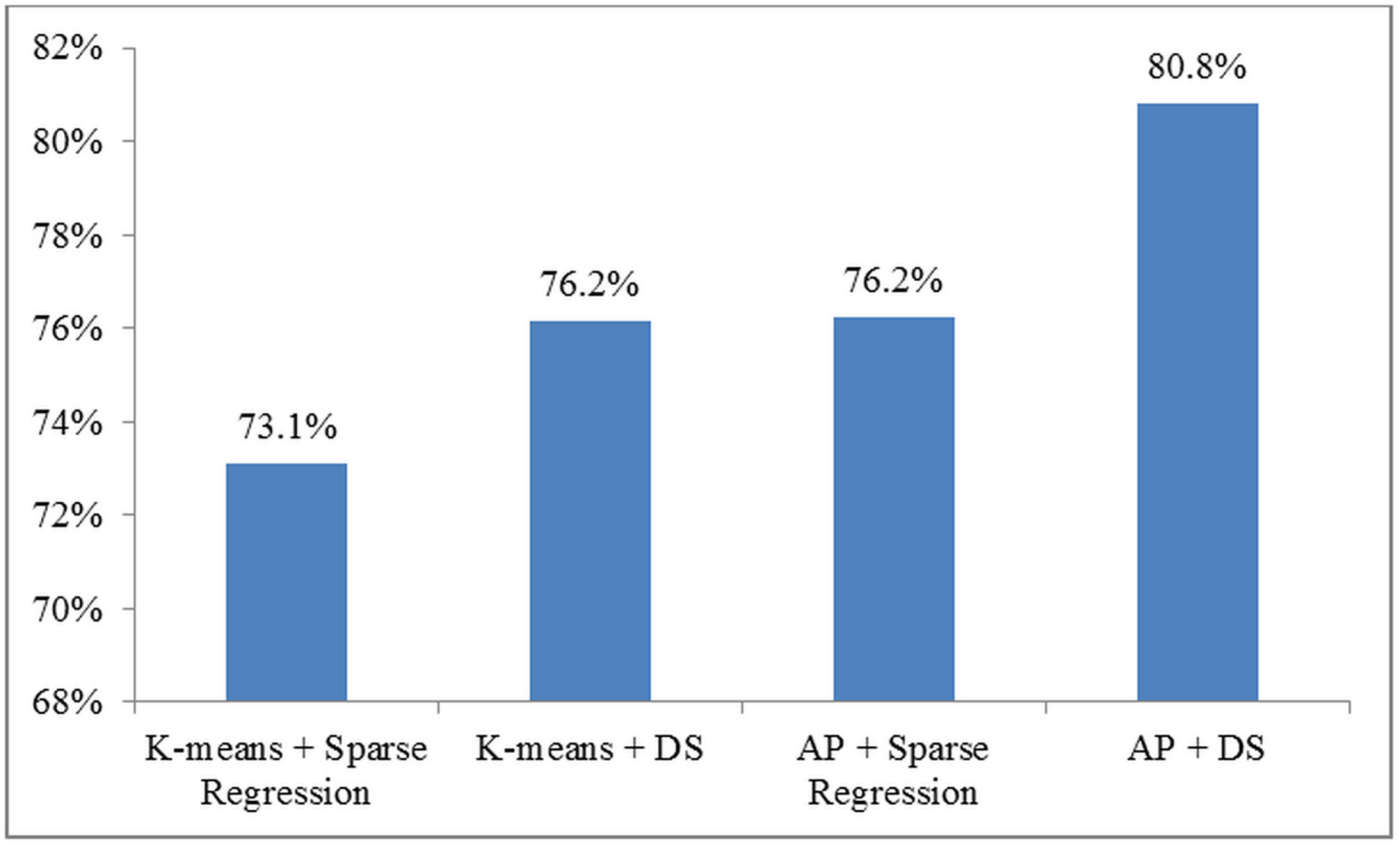
Comparison of Zhang *et al*. [8] neuroimaging marker identification and clustering with our proposed methods. Abbreviations: DS–difference statistic neuroimaging marker identification method; AP–affinity propagation clustering method. The incremental comparison shows the promise of DS and AP clustering.

Further, we successively replaced the clustering method and discriminant connection identification method in [8] with our proposed AP based multi-level clustering and difference statistic, respectively, while retaining the same classifier. For a fair comparison, the number of discriminant connections was kept fixed at 400 for both methods while all other experimental parameters were kept unchanged. The classification accuracy results obtained in this way are presented in Fig 3. The results demonstrate that the combination of our proposed AP clustering and difference statistic measure provides the best classification accuracy compared with other combinations. Additional results for other discriminant biomarker selection and classification methods are presented in S2 Table.

### Experiments with Consistent Neuroimaging Marker Identification

We experimented with varying the number of selected connections from 50 to 1,000 in steps of 50, in order to observe the dependency of classifier upon the number of selected connections. For each number of connections, the classification experiment was repeated 100 times by randomly distributing the data into training and testing subsets (50-50 split) to avoid any bias. The results presented in Table 2 show the average classification accuracy over the 100 trials for 350 to 600 connections. The complete results for 50 to 1,000 selected connections are reported in S3 Table. The results show that the classification accuracy is lower for 50 to 250 range which may point to insufficient discrimination ability of these fewer connections. The peak in classification accuracy is achieved with 450 selected connections. Afterwards, there is a slight decrease in classification accuracy but a stable trend is observed. With 450 selected connections, average accuracy of 93.1% is achieved with 95.4% sensitivity and 90.2% specificity. These results demonstrate that the proposed method is able to correctly identify sensitive neuroimaging marker which has the capability to discriminate between normal and epileptic subjects with decent accuracy. These results show that the proposed method outperforms the state-of-the-art in normal vs epileptic discrimination based on rfMRI data. We note that the proposed method is trained to classify normal and epileptic subjects at individual level, but it is not trained to discriminate between the various sub-types of epilepsy.

**Table 2:** Classification accuracy with consistent neuroimaging marker identification method.

Number of connections	Prediction accuracy	Specificity (mean)	Sensitivity (mean)
**300**	89.5% ± 2.6	85.8%	92.5%
**350**	91.4% ± 2.8	88.6%	93.6%
**400**	92.3% ± 2.4	88.8%	95.1%
**450**	93.1% ± 2.5	90.2%	95.4%
**500**	91.2% ± 2.4	88.6%	93.3%
**550**	91.5% ± 2.7	88.1%	94.3%
**600**	89.8% ± 2.7	86.1%	92.8%

For this experiment, 50% dataset is used for training and rest 50% for testing over 100 trials. It can be seen that the best accuracy is achieved with 450 connections.

## Discussion

In this section, we discuss the discriminant connections and the functional connectivity analysis with respect to brain lobes and the resting state networks.

### Discriminant Functional Connections

To better understand the neurological significance of selected connections, we studied 30 most discriminant functional connections, as listed in Table 3, which are determined on the basis of the largest absolute value in the connectivity difference matrix *D*. Among these 30 connections, 7 connections from bilaterally homologous regions have altered connectivity pattern: (i) *traverse temporal gyri*, (ii) *cuneus*, (iii) *fusiform gyri*, (iv) *inferior occipital*, (v) *precentral gyri*, (vi) *middle occipital*, and (vii) *supramarginal gyri*. Out of these 7 connections, 5 connections have decreased connectivity while 2 connections have increased connectivity in epileptic patients compared to normal subjects. These connectivity alterations may affect the auditory processing (traverse temporal gyri), visual processing (cuneus, inferior occipital, middle occipital), voluntary motor function (precentral gyri), language perception (supramarginal gyri), and recognition (fusiform gyri). In total, 17 inter-hemispheric connections are altered while only 6 connections within left hemi-sphere and 7 connections within right hemi-sphere are altered. Out of the 17 inter-hemispheric connections, 5 connections have increased connectivity while 12 connections have decreased connectivity. This indicates greater alteration due to epilepsy in inter-hemispheric connections and bilaterally homologous connections compared with healthy controls. In Ji et al. [17], the authors also identified connectivity alterations in bilaterally homologous cuneus and supramarginal gyri regions.

**Table 3:** The 30 most discriminant connections identified–the connections are sorted with respect to the corresponding absolute value in the connectivity difference matrix *D*.

S#	Connection		
	Region	Region	*D* value
1	*superior temporal pole-R*	*posterior cingulate gyrus-L*	0.197
2*	*transverse temporal gyri-R*	*transverse temporal gyri-L*	−0.188
3	inferior temporal gyrus-R	middle frontal gyrus, orbital part-R	0.183
4*	*cuneus-R*	*cuneus-L*	−0.182
5	*lingual gyrus-L*	*calcarine sulcus-R*	−0.176
6	superior frontal gyrus, medial orbital part-R	superior frontal gyrus, orbital part-R	0.162
7	gyrus rectus-R	olfactory cortex-R	−0.162
8	*orbital part of inferior frontal gyrus-L*	*area triangularis-L*	0.161
9	amygdala-R	hippocampus-R	−0.159
10	superior temporal pole-R	rolandic operculum-R	−0.158
11	*parahippocampal gyrus-R*	*hippocampus-L*	−0.158
12	*superior temporal gyrus-R*	*rolandic operculum-L*	−0.151
13	globus pallidus-L	caudate nucleus-L	−0.149
14	middle temporal gyrus-R	superior frontal gyrus, medial part-R	−0.147
15*	*middle occipital-R*	*middle occipital-L*	0.146
16*	*supramarginal gyrus-R*	*supramarginal gyrus-L*	0.144
17	*middle occipital-L*	*middle frontal gyrus*, *lateral part-R*	−0.144
18*	*fusiform gyrus-R*	*fusiform gyrus-L*	−0.142
19*	*inferior occipital-R*	*inferior occipital-L*	−0.141
20	amygdala-L	superior frontal gyrus, orbital part-L	0.140
21	*gyrus rectus-L*	*olfactory cortex-R*	−0.139
22	orbital part of inferior frontal gyrus-L	superior frontal gyrus, dorsolateral-L	0.138
23	superior temporal pole-L	superior temporal gyrus-L	−0.138
24	supramarginal gyrus-L	posterior cingulate gyrus-L	0.138
25	*middle frontal gyrus*, *orbital part-L*	*precentral gyrus-R*	0.137
26	*area triangularis-R*	*opercular part of inferior frontal gyrus-L*	−0.137
27	*insula-R*	*rolandic operculum-L*	−0.136
28	*inferior occipital-L*	*calcarine sulcus-R*	0.135
29*	*precentral gyrus-R*	*precentral gyrus-L*	−0.134
30	middle temporal pole-R	middle frontal gyrus, lateral part-R	0.133

The positive sign of the *D* value represents increased connectivity in epilepsy patients while the negative sign represents decreased connectivity in epilepsy patients. Among these 30 connections, 17 are inter-hemispheric (i.e. between left and right hemi-spheres) which are highlighted in italic font. Out of these 17 connections, total 7 connections are between bilaterally homologous brain regions which are highlighted by * in the serial column. Abbreviations: L–left hemi-sphere, R–right hemi-sphere.

### Functional Connectivity Alteration in Brain Lobes

**Fig 4:**
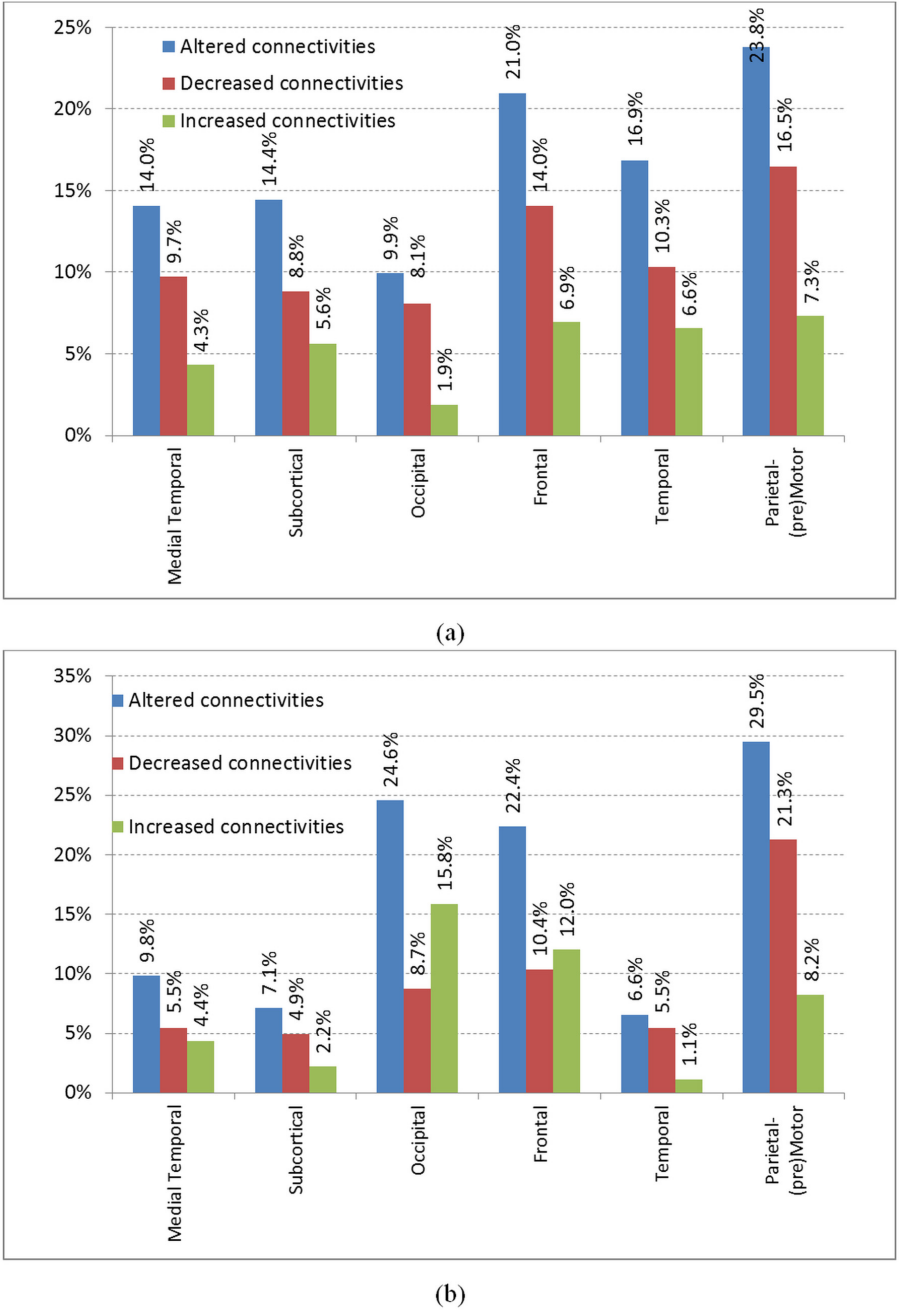
Summary of brain lobes functional alterations: (a) inter-group and (b) intra-group. For this analysis, the brain is considered to be made up of six lobes as suggested by Salvador *et al*. [31].

An important aim of this work is to study the functional alteration, either increased or decreased, in brain functional connectivity. Here, we discuss our findings in terms of systematic groups of brain regions from rfMRI BOLD signals of 90 cortical regions suggested by Salvador *et al*. [31] based upon hierarchical cluster analysis. The work of Salvador et al. found lobe groups that consist of: (i) medial temporal lobe, (ii) subcortical lobe, and the four standard neocortical lobes which are (iii) occipital lobe, (iv) frontal lobe, (v) temporal lobe, and (vi) parietal (pre) motor lobe. Though these lobes have anatomical association, it may be noted that these lobes were determined by Salvador *et al*. [31] through functional analysis from rfMRI scans of healthy controls. Since the highest classification accuracy of 93.08% is achieved using the top 450 connections, the connectivity alteration analysis is conducted on the basis of considering them as the discriminant connections, and analyzing the connections by mapping each brain cortical region [27] to a particular lobe [31]. We study the increased, decreased, or total altered inter-group and intra-group connectivity of lobes due to epilepsy, with respect to these selected connections, as shown in Fig 4.

We can infer the following findings from these results:

- Parietal-(pre) motor is most affected in terms of both inter-group and intra-group functional connectivity. In both kinds of connectivity analyses, the parietal-(pre) motor lobe is found to have reduced connectivity for epileptic subjects as compared to normal subjects. The parietal (pre) motor lobe is actively involved in movement intention and motor awareness [32], thus indicating that movement may be affected in epileptic subjects.
- Inter-lobe connectivity: the functional connectivity of all lobes to all the other lobes is decreased in epileptic subjects. This indicates a lack of synchronization across the brain regions.
- Intra-lobe alterations: the occipital and frontal lobes have increased intra-group connectivity while other lobes face decreased intra-group connectivity. The increased connectivity in the occipital and frontal lobes indicates an over-activity of the visual and cognition functions for the epileptic subjects. Earlier work by Liao et al. [29] found altered connectivity within medial temporal, frontal and parietal lobes.

### Functional Connectivity Alteration in Resting State Networks (RSNs)

**Fig 5:**
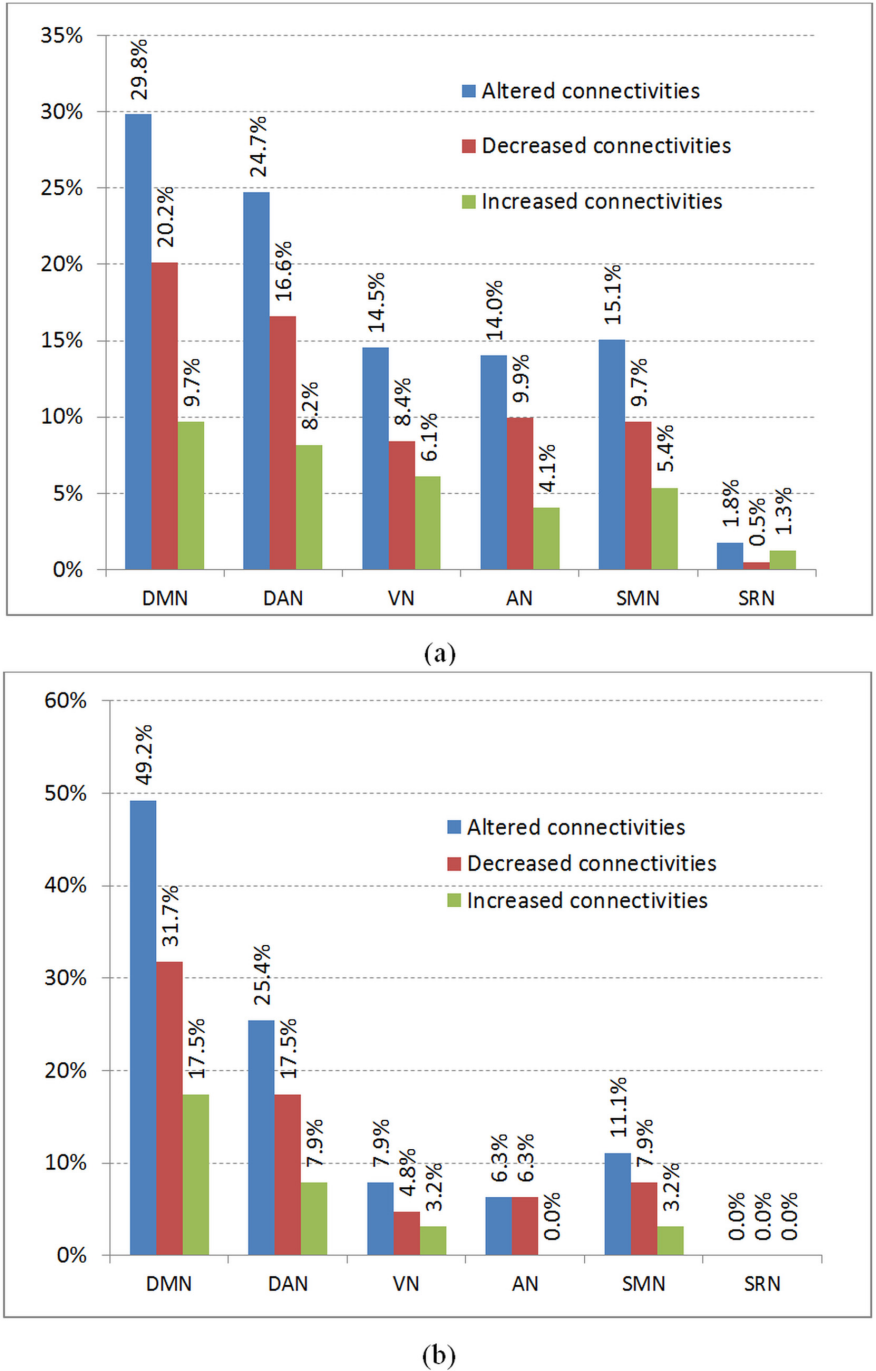
Summary of brain resting-state networks (RSNs) functional alterations: (a) inter-RSN and (b) intra-RSN. For this analysis, the brain is considered to be made up of six RSNs as suggested by Mantini *et al*. [33]. Abbreviations: DMN–default mode network; DAN–dorsal attention network; VN–visual network; AN: auditory network; SMN: somato motor network; SRN: self-referential network.

Lastly, we analyze the functional connectivity alteration, either increased or decreased, in the brain resting state networks (RSNs) suggested by Mantini *et al*. [33]. The six RSNs are listed as: DMN–default mode network; DAN–dorsal attention network; VN–visual network; AN: auditory network; SMN: somato motor network; SRN: self-referential network. Similar to the lobe analysis in the above section, the RSNs analysis is also conducted using the 450 discriminant connections which yielded the classification accuracy of 93.08%. We studied the functional connectivity alteration, either increased or decreased, caused by epilepsy in terms of these six RSNs. The functional connectivity alterations are categorized with respect to inter-RSN and intra-RSN, as shown in Fig 5.

The connectivity alteration results clearly show that the DMN is affected the most with respect to inter-RSN and intra-RSN in terms of both increased and decreased connectivity. This pattern of serious alteration is also followed by DAN which is the second highest affected RSN, both in inter-RSN and intra-RSN analysis with respect to increased and decreased connectivity. Our results confirm the earlier results reported in the previous studies [14, 15, 29, 34] that epilepsy affects DMN and DAN. This indicates that epilepsy may impair the brain internal processing and attention systems of an individual. We believe that this impairment in DMN and DAN is contributed by functional defects exhibited by patients suffering from epilepsy.

### Limitations

The proposed work considered the generalized and focal sub-types of epilepsy as one common group to learn the classification model and to perform the subsequent connectivity analysis. For a fair comparison with Zhang et al. [8], we followed the similar protocol of grouping the sub-types together. Moreover, unlike Zhang et al., the proposed method does not extract any biomarker which measures the asymmetry across brain hemi-spheres which is a characteristic that can be present in generalized vs focal epilepsy studies. Thus, the proposed method is independent of this bias. However, due to the underlying differences in pathophysiology and the nature of hemi-sphere specific characteristics, there is indeed interest to consider the sub-types as different groups which may be considered in a future study. This will need the scan acquisition of a sufficient number of subjects available in each group.

In terms of classification, the proposed method is able to predict with decent accuracy from rfMRI scan whether a given individual scan is from a normal or an epileptic subject. However, the classifier in our method is not trained to further predict the severity of epilepsy or the specific sub-type of epilepsy for an individual. We believe that such a method can be developed with the availability of a larger amount of training data for each sub-type and the availability of ground truth about the severity level.

In this work, the automated anatomical labeling (AAL) template of Tzourio-Mazoyer et al. [27] was used to select a representative group of the Volume of Interest (VOI) regions rather than conducting the voxel-wise analysis which would be computationally exhaustive and difficult to interpret. Further, the AAL template has been used extensively over the last decade for functional analysis since the cortical regions from AAL template can be mapped to functional networks (e.g. resting state networks [33]). An alternative region selection scheme can be based upon functional parcellation, for example the recently proposed parcellation of Gordon et al. [35]. In our view, each parcellation scheme has its own pros and cons. For example, the functional parcellation scheme of Gordon et al. is entirely functional specific and may not identify any anatomical related functional problems caused by a mental condition [35]. In this respect, the functional activity can be considered to be the temporal characteristics exhibited by the BOLD signal. The analysis of this functional activity is still possible despite the AAL template, since the AAL template is used primarily for the selection of VOI regions.

## Conclusions

In this paper, we studied the problem of connectivity analysis of epilepsy patients from rfMRI scans. For this purpose, a novel method was proposed for estimation of functional connectivity between brain regions. The proposed clustering method allowed the estimation of brain community matrix. Our results demonstrate that this community matrix contains crucial information which can be essentially employed to discriminate epileptic subjects from normal subjects. This community matrix further enabled the extraction of discriminant connections using a novel difference statistic measure. These discriminant connections were then utilized to train an automated SVM classification method which can accurately predict whether a given rfMRI scan belongs to epilepsy or normal class. The proposed method achieved a promising classification accuracy of 93.08%, in comparison to 80.20% accuracy reported by Zhang *et al*. [8]. The connectivity analysis on brain resting state networks (RSNs) confirms that epilepsy impairs the performance of default mode network (DMN) and dorsal attention network (DAN). In terms of lobe connectivity, the parietal (pre) motor lobe demonstrates decreased connectivity for epilepsy patients which may hint towards impaired movement intent and motion awareness in epilepsy subjects. The approach proposed in this paper is not only able to identify the altered connections, but also utilizes them to achieve relatively high classification accuracy. The proposed algorithm can be applied to study other neurological disorders like schizophrenia and Alzheimer’s disease.

## Supporting Information

S1 Table The list of cortical regions as per automated anatomically labelled (AAL) template of Tzourio-Mazoyer *et al*. [27]. The list shows 45 regions, which exist in both left and right brain hemi-spheres thus yielding a total of 90 cortical regions.
(DOCX)

S2 Table Classification accuracy by experimenting with other common methods for discriminant biomarker selection and a popular classifier (random forest). (DOCX)

S3 Table Classification accuracy with consistent neuroimaging marker identification method by variation in number of selected connections. (DOCX)

## References

[1] Van Den Heuvel MP, Hulshoff Pol HE. Exploring the brain network: a review on resting-state fMRI functional connectivity. European Neuropsychopharmacology. 2010;20(8):519–34. 10.1016/j.euroneuro.2010.03.008 20471808

[2] Biswal B, Zerrin Yetkin F, Haughton VM, Hyde JS. Functional connectivity in the motor cortex of resting human brain using echo-planar mri. Magnetic resonance in medicine. 1995;34(4):537–41. 8524021 10.1002/mrm.1910340409

[3] Li K, Guo L, Nie J, Li G, Liu T. Review of methods for functional brain connectivity detection using fMRI. Computerized Medical Imaging and Graphics. 2009;33:131–9. 10.1016/j.compmedimag.2008.10.011 19111443 PMC2724763

[4] Lee MH, Hacker CD, Snyder AZ, Corbetta M, Zhang D, Leuthardt EC, et al Clustering of resting state networks. PloS one. 2012;7(7):e40370 10.1371/journal.pone.0040370 22792291 PMC3392237

[5] Dey S, Rao R, Shah M. Exploiting the Brain's Network Structure in Identifying ADHD Subjects. Frontiers in System Neuroscience. 2012;6. 10.3389/fnsys.2012.00075 PMC3499771 23162440

[6] Tao H, Guo S, Ge T, Kendrick KM, Xue Z, Liu Z, et al Depression uncouples brain hate circuit. Molecular psychiatry. 2011;18(1):101–11. 10.1038/mp.2011.127 21968929 PMC3526729

[7] Zhang D, Raichle ME. Disease and the brain's dark energy. Nature Reviews Neurology. 2010;6(1):15–28. 10.1038/nrneurol.2009.198 20057496

[8] Zhang J, Cheng W, Wang Z, Zhang Z, Lu W, Lu G, et al Pattern classification of large-scale functional brain networks: identification of informative neuroimaging markers for epilepsy. PloS one. 2012;7(5):e36733 10.1371/journal.pone.0036733 22615802 PMC3355144

[9] Venkataraman A, Kubicki M, Westin CF, Golland P, editors. Robust feature selection in resting-state fMRI connectivity based on population studies. IEEE Computer Society Conference on Computer Vision and Pattern Recognition Workshops (CVPRW); 2010 13–18 June 2010. 10.1109/CVPRW.2010.5543446 PMC3110085 21660131

[10] Koch W, Teipel S, Mueller S, Benninghoff J, Wagner M, Bokde AL, et al Diagnostic power of default mode network resting state fMRI in the detection of Alzheimer's disease. Neurobiology of Aging. 2012;33(3):466–78. 10.1016/j.neurobiolaging.2010.04.013 20541837

[11] Greicius MD, Srivastava G, Reiss AL, Menon V. Default-mode network activity distinguishes Alzheimer's disease from healthy aging: evidence from functional MRI. Proceedings of the National Academy of Sciences. 2004;101(13):4637–42. 10.1073/pnas.0308627101 PMC384799 15070770

[12] Tian L, Jiang T, Liang M, Zang Y, He Y, Sui M, et al Enhanced resting-state brain activities in ADHD patients: a fMRI study. Brain and Development. 2008;30(5):342–8. 18060712 10.1016/j.braindev.2007.10.005

[13] Organization WH. Neurological Disorder: Public Health Challenges. Geneva: WHO Press; 2006.

[14] Zhang Z, Lu G, Zhong Y, Tan Q, Yang Z, Liao W, et al Impaired attention network in temporal lobe epilepsy: A resting FMRI study. Neuroscience Letters. 2009;458(3):97–101. 10.1016/j.neulet.2009.04.040. 10.1016/j.neulet.2009.04.040 19393717

[15] Haneef Z, Lenartowicz A, Yeh HJ, Engel J Jr, Stern JM. Effect of lateralized temporal lobe epilepsy on the default mode network. Epilepsy & Behavior. 2012;25(3):350–7. 23103309 10.1016/j.yebeh.2012.07.019 PMC4209897

[16] Ji G-J, Zhang Z, Zhang H, Wang J, Liu DQ, Zang Y-F, et al Disrupted causal connectivity in mesial temporal lobe epilepsy. 2013;8(5):e63183 10.1371/journal.pone.0063183 23696798 PMC3655975

[17] Ji G-J, Zhang Z, Xu Q, Zang Y-F, Liao W, Lu G. Generalized tonic-clonic seizures: aberrant interhemispheric functional and anatomical connectivity. Radiology. 2014;271(3):839–47. 10.1148/radiol.13131638 24588676

[18] Chen S, Wu X, Lui S, Wu Q, Yao Z, Li Q, et al Resting-state fMRI study of treatment-naïve temporal lobe epilepsy patients with depressive symptoms. NeuroImage. 2012;60(1):299–304. 10.1016/j.neuroimage.2011.11.092 22178816

[19] Zung W. A self-rating scale for depression. Archives of General Psychiatry. 12:63–70. 14221692 10.1001/archpsyc.1965.01720310065008

[20] Frey BJ, Dueck D. Clustering by Passing Messages Between Data Points. Science. 2007;315:972–6. 17218491 10.1126/science.1136800

[21] Zhang J, Li D, Chen H, Fang F. Analysis of activity in fMRI data using affinity propagation clustering. Computer Methods in Biomechanics and Biomedical Engineering. 2011;14(03):271–81. 21347914 10.1080/10255841003766829

[22] Jiang Z, Huafu C, editors. Analysis of activity in fMRI data for multitask experimental paradigm using affinity propagation clustering. The 2nd International Conference on Computer and Automation Engineering (ICCAE); 2010 26–28 Feb. 2010.

[23] Liu D, Lu W, Zhong N. Clustering of fMRI Data Using Affinity Propagation In: Yao Y, Sun R, Poggio T, Liu J, Zhong N, Huang J, editors. Brain Informatics. Lecture Notes in Computer Science 6334: Springer Berlin Heidelberg; 2010 p. 399–406.

[24] Chao-Gan Y, Yu-Feng Z. DPARSF: a MATLAB toolbox for “pipeline” data analysis of resting-state fMRI. Frontiers in Systems Neuroscience. 2010;4. 10.3389/fnsys.2010.00013 PMC2889691 20577591

[25] Friston KJ. Statistical parametric mapping Neuroscience Databases Springer; 2003 p. 237–50.

[26] Song X-W, Dong Z-Y, Long X-Y, Li S-F, Zuo X-N, Zhu C-Z, et al REST: a toolkit for resting-state functional magnetic resonance imaging data processing. PloS one. 2011;6(9):e25031 10.1371/journal.pone.0025031 21949842 PMC3176805

[27] Tzourio-Mazoyer N, Landeau B, Papathanassiou D, Crivello F, Etard O, Delcroix N, et al Automated anatomical labeling of activations in SPM using a macroscopic anatomical parcellation of the MNI MRI single-subject brain. NeuroImage. 2002;15(1):273–89. 11771995 10.1006/nimg.2001.0978

[28] Zhang Z, Liao W, Chen H, Mantini D, Ding J-R, Xu Q, et al Altered functional–structural coupling of large-scale brain networks in idiopathic generalized epilepsy. Brain. 2011;134(10):2912–28. 21975588 10.1093/brain/awr223

[29] Liao W, Zhang Z, Pan Z, Mantini D, Ding J, Duan X, et al Altered functional connectivity and small-world in mesial temporal lobe epilepsy. PloS one. 2010;5(1):e8525 10.1371/journal.pone.0008525 20072616 PMC2799523

[30] Cortes C, Vapnik V. Support vector machine. Machine Learning. 1995;20(3):273–97.

[31] Salvador R, Suckling J, Coleman MR, Pickard JD, Menon D, Bullmore E. Neurophysiological architecture of functional magnetic resonance images of human brain. Cerebral Cortex. 2005;15(9):1332–42. 15635061 10.1093/cercor/bhi016

[32] Desmurget M, Sirigu A. A parietal-premotor network for movement intention and motor awareness. Trends in Cognitive Sciences. 2009;13(10):411–9. 10.1016/j.tics.2009.08.001. 10.1016/j.tics.2009.08.001 19748304

[33] Mantini D, Perrucci MG, Del Gratta C, Romani GL, Corbetta M. Electrophysiological signatures of resting state networks in the human brain. Proceedings of the National Academy of Sciences. 2007;104(32):13170–5. 10.1073/pnas.0700668104 PMC1941820 17670949

[34] Liao W, Zhang Z, Pan Z, Mantini D, Ding J, Duan X, et al Default mode network abnormalities in mesial temporal lobe epilepsy: a study combining fMRI and DTI. Human brain mapping. 2011;32(6):883–95. 10.1002/hbm.21076 20533558 PMC6870458

[35] Gordon EM, Laumann TO, Adeyemo B, Huckins JF, Kelley WM, Petersen SE. Generation and evaluation of a cortical area parcellation from resting-state correlations. Cerebral Cortex. 2014:bhu239 25316338 10.1093/cercor/bhu239 PMC4677978

