# Supplementary material for: Functional Connectivity Alterations in Epilepsy from Resting-State Functional MRI
Source: PLoS One. 2015 Aug 7;10(8):e0134944. doi: 10.1371/journal.pone.0134944 (PMC4529140; doi:10.1371/journal.pone.0134944)
Supplement: S1 Table — The list shows 45 regions, which exist in both left and right brain hemi-spheres thus yielding a total of 90 cortical regions. (DOCX) [file pone.0134944.s001.docx]

**S1 Table: The list of cortical regions as per automated anatomically labelled (AAL) template of Tzourio-Mazoyer *et al.* [27]. The list shows 45 regions, which exist in both left and right brain hemi-spheres thus yielding a total of 90 cortical regions.**

| **No** | **Region Name** | **No** | **Region Name** |
| --- | --- | --- | --- |
| 1 | Precentral gyrus | 24 | Lingual gyrus |
| 2 | Superior frontal gyrus, dorsolateral | 25 | Superior occipital |
| 3 | Superior frontal gyrus, orbital part | 26 | Middle occipital |
| 4 | Middle frontal gyrus (lateral part) | 27 | Inferior occipital |
| 5 | Middle frontal gyrus, orbital part | 28 | Fusiform gyrus |
| 6 | Opercular part of inferior frontal gyrus | 29 | Postcentralgyrus |
| 7 | Triangularis part of inferior frontal gyrus | 30 | Superior parietal lobule |
| 8 | Orbital part of inferior frontal gyrus | 31 | Inferior parietal lobule |
| 9 | Rolandic operculum | 32 | Supramarginalgyrus |
| 10 | Supplementary motor area | 33 | Angular gyrus |
| 11 | Olfactory cortex | 34 | Precuneus |
| 12 | Superior frontal gyrus, medial part | 35 | Paracentral lobule |
| 13 | Superior frontal gyrus, medial orbital part | 36 | Caudate nucleus |
| 14 | Gyrus rectus | 37 | Putamen |
| 15 | Insula | 38 | Globuspallidus |
| 16 | Anterior cingulate gyrus | 39 | Thalamus |
| 17 | Middle cingulate | 40 | Transverse temporal gyri |
| 18 | Posterior cingulate gyrus | 41 | Superior temporal gyrus |
| 19 | Hippocampus | 42 | Superior temporal pole |
| 20 | Parahippocampalgyrus | 43 | Middle temporal gyrus |
| 21 | Amygdala | 44 | Middle temporal pole |
| 22 | Calcarine sulcus | 45 | Inferior temporal gyrus |
| 23 | Cuneus |  |  |
