# Supplementary material for: Functional Connectivity Alterations in Epilepsy from Resting-State Functional MRI
Source: PLoS One. 2015 Aug 7;10(8):e0134944. doi: 10.1371/journal.pone.0134944 (PMC4529140; doi:10.1371/journal.pone.0134944)
Supplement: S2 Table — (DOCX) [file pone.0134944.s002.docx]

**S2 Table: Classification accuracy by experimenting with other common methods for discriminant biomarker selection and a popular classifier (random forest).**

| No | Feature selection | Classifier | Prediction accuracy |
| --- | --- | --- | --- |
| 1 | Difference statistic | SVM | $80.8\%$ |
| 2 | Difference statistic | RF | $76.9\%$ |
| 3 | Gini index | SVM | $80.1\%$ |
| 4 | Gini index | RF | $78.3\%$ |
| 5 | t-test | SVM | $78.9\%$ |
| 6 | Wilcoxon | SVM | $72.4\%$ |

Evaluation of different feature selection methods and classifiers. Number of selected connections is fixed to 400 to make comparison with Zhang et al. Different biomarker selection methods are employed while the difference statistic yields the highest accuracy. Data split for training and testing is according to Table 1. We employed cross validation using 100 trials for assessing classifier performance. RF = Random Forest, SVM = Support Vector Machine.
