# Supplementary material for: Functional Connectivity Alterations in Epilepsy from Resting-State Functional MRI
Source: PLoS One. 2015 Aug 7;10(8):e0134944. doi: 10.1371/journal.pone.0134944 (PMC4529140; doi:10.1371/journal.pone.0134944)
Supplement: S3 Table — (DOCX) [file pone.0134944.s003.docx]

**S3 Table: Classification accuracy with consistent neuroimaging marker identification method by variation in number of selected connections.** The number of selected connections is varied from 50 to 1,000 and the classification performance is studied.

| Number of connections | Prediction accuracy | Specificity (mean) | Sensitivity (mean) |
| --- | --- | --- | --- |
| $\boldsymbol{50}$ | $76.9\%\pm3.9$ | $73.2\%$ | $79.8\%$ |
| $\boldsymbol{100}$ | $80.4\%\pm3.5$ | $76.8\%$ | $83.2\%$ |
| $\boldsymbol{150}$ | $83.8\%\pm3.8$ | $80.5\%$ | $86.5\%$ |
| $\boldsymbol{200}$ | $86.3\%\pm3.1$ | $83.0\%$ | $88.9\%$ |
| $\boldsymbol{250}$ | $87.3\%\pm2.6$ | $83.7\%$ | $90.2\%$ |
| $\boldsymbol{300}$ | $89.5\%\pm2.6$ | $85.8\%$ | $92.5\%$ |
| $\boldsymbol{350}$ | $91.4\%\pm2.8$ | $88.6\%$ | $93.6\%$ |
| $\boldsymbol{400}$ | $92.3\%\pm2.4$ | $88.8\%$ | $95.1\%$ |
| $\boldsymbol{450}$ | $93.1\%\pm2.5$ | $90.2\%$ | $95.4\%$ |
| $\boldsymbol{500}$ | $91.2\%\pm2.4$ | $88.6\%$ | $93.3\%$ |
| $\boldsymbol{550}$ | $91.5\%\pm2.7$ | $88.1\%$ | $94.3\%$ |
| $\boldsymbol{600}$ | $89.8\%\pm2.7$ | $86.1\%$ | $92.8\%$ |
| $\boldsymbol{650}$ | $91.4\%\pm2.8$ | $88.4\%$ | $93.9\%$ |
| $\boldsymbol{700}$ | $91.1\%\pm2.9$ | $87.4\%$ | $94.0\%$ |
| $\boldsymbol{750}$ | $90.1\%\pm2.6$ | $86.8\%$ | $92.8\%$ |
| $\boldsymbol{800}$ | $91.1\%\pm2.6$ | $89.0\%$ | $92.8\%$ |
| $\boldsymbol{850}$ | $90.3\%\pm2.9$ | $85.4\%$ | $94.2\%$ |
| $\boldsymbol{900}$ | $90.6\%\pm2.9$ | $87.3\%$ | $93.2\%$ |
| $\boldsymbol{950}$ | $90.9\%\pm2.8$ | $87.4\%$ | $93.6\%$ |
| $\boldsymbol{1000}$ | $90.6\%\pm3.3$ | $86.7\%$ | $93.7\%$ |

For this experiment, 50% dataset is used for training and rest 50% for testing over 100 trials. It can be seen that the best accuracy is achieved with 450 connections.
